# Deciphering the Role of *Waxy* Gene Mutations in Enhancing Rice Grain Quality

**DOI:** 10.3390/foods13111624

**Published:** 2024-05-23

**Authors:** Yong Yang, Lihui Zhou, Linhao Feng, Jianying Jiang, Lichun Huang, Qing Liu, Yadong Zhang, Changquan Zhang, Qiaoquan Liu

**Affiliations:** 1 Key Laboratory of Crop Genomics and Molecular Breeding of Jiangsu Province, Zhongshan Biological Breeding Laboratory, Yangzhou University, Yangzhou 225009, China; yongyang951208@gmail.com (Y.Y.); zhoulihui@jaas.ac.cn (L.Z.); linhaofeng123@126.com (L.F.); jiangjianying2022@163.com (J.J.); lchuang@yzu.edu.cn (L.H.); qliu2102002@gmail.com (Q.L.); qqliu@yzu.edu.cn (Q.L.); 2 Jiangsu Key Laboratory of Crop Genetics and Physiology, Co-Innovation Center for Modern Production Technology of Grain Crops of Jiangsu Province, Yangzhou University, Yangzhou 225009, China; 3 Jiangsu High Quality Rice Research and Development Center, Jiangsu Key Laboratory for Agro-Biology, Institute of Food Crops, Jiangsu Academy of Agricultural Sciences, Nanjing 210014, China; zhangyd@jaas.ac.cn; 4 Commonwealth Scientific and Industrial Research Organisation Agriculture and Food, GPO Box 1700, Canberra, ACT 2601, Australia

**Keywords:** soft rice, low amylose, grain transparency, *Wx*, eating and cooking quality

## Abstract

Amylose content (AC) stands as a pivotal determinant of rice grain quality, primarily governed by the *Waxy* gene (*Wx*). The allelic variation within this gene, particularly the presence of the *Wx^mp^* allele derived from the ancestral *Wx^mq^* allele, significantly influences AC and is prevalent among soft *japonica* rice varieties in southern China. Although both alleles are associated with lower AC, there remains a paucity of detailed understanding regarding the interplay between specific functional single nucleotide polymorphisms (SNPs) within these alleles and the overarching rice grain quality. To investigate this, we engineered three distinct transgenic rice lines, each harboring the *Wx^mp^*, *Wx^mq^*, or *Wx^b−5c^* alleles in the background of the glutinous rice cultivar Nip(*wx*). This suite of transgenic rice lines showcased varying degrees of grain transparency inversely correlated to AC, which in turn influenced other physicochemical properties of the rice grains, such as taste value of cooked rice, gel consistency, and starch pasting properties. Additionally, analyses of gene expression and enzyme activity revealed that the functional SNPs, Ex4-53G to A and Ex5-53T to C, lead to a decline in the activity of granule-bound starch synthase I (GBSSI) without altering expression levels.

## 1. Introduction

Rice (*Oryza sativa* L.), a staple food for over half of the world’s population, plays a crucial role in global food security. Quality improvement remains a paramount concern for both consumers and rice breeders [1,2], with evaluation criteria spanning appearance, taste, processing characteristic, and nutritional value [3]. In particular, consumer interest peaks in attributes such as appearance, cooking quality, and taste. The endosperm, which is the edible portion of rice grains, primarily consists of starch along with proteins, lipids, and other components. Starch, as the primary storage compound in the endosperm, critically influences the taste quality of rice due to its composition and structural characteristics [4]. Starch in rice is bifurcated into two primary types: branched amylopectin, which forms the bulk of the starch content, and amylose, characterized by its long-chain linear glucose polymers. In glutinous rice, amylopectin virtually constitutes the entirety of its starch content. Conversely, amylose emerges as a pivotal component in non-glutinous rice, with amylose content (AC) critically influencing the grain’s quality attributes [5]. Typically, rice with elevated AC yields a firmer texture upon cooking, whereas varieties with diminished AC are noted for their softness and stickiness [6]. Thus, varieties manifesting AC levels ranging from 8% to 13%, bridging the gap between glutinous and sticky rice, are often categorized as soft rice. These varieties are distinguished by their tender and cohesive texture, coupled with superior taste and palatability [7].

The granule-bound starch synthase I (GBSSI), encoded by the *Waxy* (*Wx*) gene, plays a crucial role in amylose synthesis within rice endosperm. The genetic diversity in AC across rice varieties is closely linked to allelic variations in the *Wx* gene, highlighting its significant impact on rice quality [8]. To date, researchers have identified at least nine *Wx* alleles, namely *Wx^lv^*, *Wx^a^*, *Wx^in^*, *Wx^b^*, *Wx^mw^*, *Wx^mp^*, *Wx^mq^*, *Wx^op^*, and *wx*, each contributing uniquely to the starch composition of rice [8,9]. The *Wx^mp^* allele, in particular, has gained prominence in the breeding of soft rice varieties in south China, leading to the popularization of *japonica* varieties such as Nangeng9108, and Huruan 1212. Originating from the low-AC *japonica* cultivar Milky Princess, the *Wx^mp^* allele is characterized by a G to A mutation in exon 4 (Ex4-53A), resulting in the substitution of Arg158 by His158, distinguishing it from the *Wx^b^* allele [10]. Milky Queen, a sibling line of Milky Princess, exhibits an additional mutation, featuring the *Wx^mq^* allele, a T to C mutation resulting in the substitution of Tyr191 by His191 in exon 5 (Ex5-52C), further diversifying the allelic variations from *Wx^b^* [11]. This nuanced genetic variation has, at times, led to confusion among breeders regarding the distinct contributions of the *Wx^mp^* and *Wx^mq^* alleles to rice grain quality. The dynamic interaction between these two functional alleles and their synergistic influence on rice grain phenotypes still requires thorough investigation for a clearer understanding.

**Figure 1:**
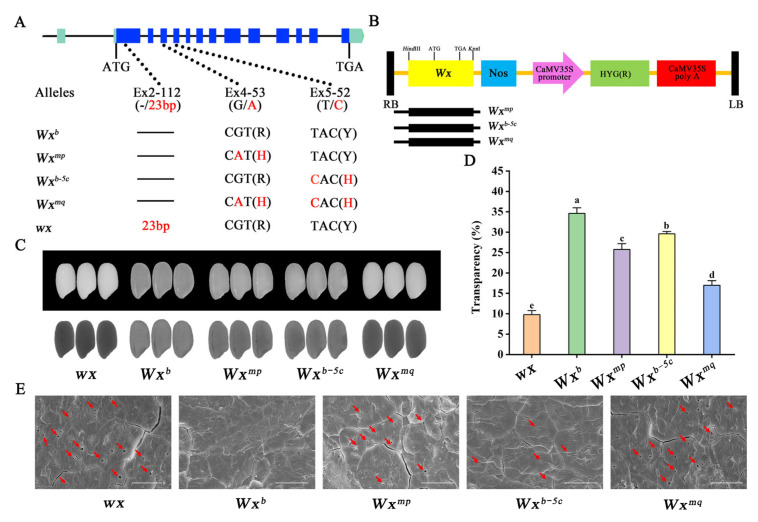
Characterization of grain appearance quality in transgenic rice lines. (**A**) Depicts the structural organization and allelic diversity of *Wx* gene, highlighting the genetic variations that underpin starch synthesis and ultimately affect grain quality. (**B**) Illustrates the configurations of transgenic vectors, each incorporating genomic DNA segments specific to the *Wx^mp^*, *Wx^b^*^−*5c*^, and *Wx^mq^* alleles. (**C**) A visual comparison of grain appearance under two lighting conditions: reflected light (upper panel) and transmitted light (down panel). (**D**) Grain transparency. (**E**) Starch morphology in transverse sections of rice grains. Arrows indicate the position of holes, Scale bar, 5 μm. Statically significant differences are denoted by different letters (*p* < 0.01).

In this study, we engineered complementary vectors encapsulating the complete genomic DNA sequences of the *Wx^mp^* (Ex4-53A), *Wx^mq^* (Ex4-53A + Ex5-52C), and *Wx^b−5c^* (Ex5-52C) alleles. These vectors were then introduced into the glutinous rice variety Nip(*wx*), creating a near-isogenic line (NIL) devoid of the *wx* allele in the *japonica* rice Nipponbare (NIP) background, which naturally carries the *Wx^b^* allele (Figure 1A,B and Figure S1). Through a systematic evaluation of the grain quality attributes of the resulting homozygous transgenic rice lines, we elucidated the impacts of the two functional sites on amylose synthesis, grain morphology, the sensory qualities of cooked rice, and the physicochemical properties of starch. This research presents valuable genetic materials and lays a conceptual foundation for the development and enhancement of rice varieties characterized by moderate AC and superior culinary quality.

## 2. Materials and Methods

### 2.1. Plant Materials and Growth Conditions

The experimental materials used in this study comprised the *japonica* cultivar Nipponbare (Nip), which harbors the *Wx^b^* allele, and its near-isogenic line (NIL) within the Nip background, referred to as Nip(*wx*) and containing the null *wx* allele. Further, transgenic rice lines—Nip(*wx*)-*Wx^mp^*, Nip(*wx*)-*Wx^b^*^−*5c*^, and Nip(*wx*)-*Wx^mq^*—were generated against the Nip(*wx*) background. These specific rice lines were cultivated at the experimental farm of Yangzhou University (Yangzhou, China, 32°23′ N) during the 2022 growing season. The experimental design included planting 20 seedlings for each strain, with the replication of three biological repeats. Consistent field management practices were implemented across all variants.

### 2.2. Plasmids and Rice Transformation

PCR amplification was conducted using genomic DNA from the *Japonica* cultivars Nip and NJ46 (carrying the *Wx^mp^* allele) as templates. This process employed primers QWx-1, QWx-2, QWx-3, and QWx-4 (Table S1), yielding two distinct fragments corresponding to the *Wx^b^* and *Wx^mp^* alleles, with lengths of 4 kb and 4.4 kb, respectively (Figure S1). The PCR reactions were performed in a 50 µL volume using PrimeSTAR^®^ HS DNA Polymerase (TaKaRa, Kyoto, Japan). The amplification conditions were as follows: an initial denaturation at 94 °C for 5 min, followed by 36 cycles of denaturation at 98 °C for 10 s and annealing/extension at 68 °C for 4 min, concluding with a final extension at 72 °C for 5 min. For the generation of the *Wx^b−5c^* and *Wx^mq^* expression vectors, genomic DNA from Nip and NJ46 was used as templates. Initially, the 1st DNA segment was amplified with primers QWx-1/QWx-2 from both Nip and NJ46 genomic DNA separately. Subsequently, a DNA segment bearing a T to C mutation in exon 5 was amplified using NJ46 genomic DNA and primers QWx-3/QWx-5C-R and QWx-5C-F/QWx-4 (Table S1), resulting in the 2nd DNA segment. This DNA segment was then ligated with the 1st DNA segment amplified from Nip to assemble the *Wx^b−5c^* segments. Similarly, the 2nd DNA segment was ligated with the 1st DNA segment amplified from NJ46 to construct the *Wx^mq^* allele. The resultant PCR products were cloned into the pCAMBIA1300 vector (Cambia, Canberra, Australia). The constructed plasmids, namely PC1300-*Wx^mp^*, PC1300-*Wx^b−5c^*, and PC1300-*Wx^mq^* (Figure 1B), were introduced into *Agrobacterium tumefaciens* strain EHA105. This was followed by the transformation into Nip(*wx*) via agrobacterium-mediated transformation [12]. The identification of transgene copy number was performed according to the method established by Ding et al. [13], employing 10 ng of genomic DNA as a PCR template along with *Wx*-specific primers and an endogenous single-copy molecular marker (Table S1).

### 2.3. Observation of Grain Transparency

To evaluate grain transparency, photographs of various samples of white rice were captured under transmitted light conditions. These color images were subsequently converted into black and white for analysis. The transparency rate (%) was determined by calculating the ratio of the average grain grayscale value of the image background. The analysis was performed using the software ImageJ 1.54i (available at https://imagej.net/, accessed on 4 March 2024), following the methodology described by Zhang et al. [14].

### 2.4. Examination of Cavities within Starch Granules

To observe the internal structure of starch granules, rice grains were subjected to rapid freezing in liquid nitrogen. Subsequently, these frozen samples were cut to produce cross-sections for microscopic examination. The prepared cross-sections were affixed to aluminum specimen stubs using dual adhesive tabs, followed by sputter-coating with gold to enhance electron conductivity and image clarity. The samples were then examined under an S-4800 environmental scanning electron microscopy (SEM) instrument (Hitachi, Tokyo, Japan) to visualize the microstructural details of the starch granules.

### 2.5. Grain Quality Analyses

The apparent amylose content (AAC) of milled rice flour was determined using an iodine colorimetric method. For this assay, 20.0 mg of rice flour was weighed into a 2 mL centrifuge tube, to which 0.1 mL of ethanol was added. Then, 1.8 mL of 1 N NaOH solution was added, and the suspension was thoroughly mixed. The mixture was then incubated at 60 °C with shaking at 200 rpm for 2 h. Subsequently, 100 µL of the suspension was transferred to a 10 mL centrifuge tube containing 9 mL distilled water (dH_2_O), 200 µL sodium acetate solution (pH 4.3), and 200 µL 0.02% iodine solution. The resulting mixture was vigorously stirred, and the absorbance was measured at 620 nm. AAC was calculated using a standard curve prepared with amylose standards. The method for determining gel consistency (GC) involved weighing 100.0 mg of rice flour into a 13 mm × 150 mm tube. To each tube, 0.2 mL of 95% ethanol containing 0.025% thymol blue was added, along with 2 mL of 1N KOH. After thoroughly mixing, the tubes were placed in a vigorously boiling water bath for 8 min. After being removed from the water bath, the tubes were allowed to stand at ambient temperature for 5 min and then cooled in an ice water bath for 20 min. The tubes were subsequently laid horizontally on a light box over graph paper, and after 1 h, the distance migrated by the gel in the tube was measured. Rice taste value was assessed using the Cooked Rice Taste Analyzer (CRTA) Model STA1B (Sake, Hiroshima, Japan). Approximately 30 g of milled rice grains were soaked in water in an aluminum container for 30 min, then rinsed three times with cold water. The rice grains were then subjected to steaming with a rice-to-water weight ratio of 1:1.2 for 30 min and were maintained in a warmed state for 10 min prior to being cooled to ambient temperature before analysis.

### 2.6. Measurement of the Gelatinization and Pasting Properties of Rice

The determination of gelatinization temperatures was executed employing a differential scanning calorimeter (DSC) model 200 F3 (Netzsch Instruments North America Llc, Burlington, MA, USA). The analysis yielded DSC curves that elucidated various parameters such as onset (To), peak (Tp), and conclusion (Tc) temperatures, along with the enthalpy of gelatinization (ΔH), which indicates the thermal energy required for the gelatinization process. Furthermore, the pasting properties of rice were determined using a Rapid Visco-Analyzer (RVA) (Techmaster, Newport Scientific, Warriewood, Australia) following the method described by Zhang et al. [15].

### 2.7. Gel Permeation Chromatograms

Isoamylase (EC 3.2.1.68, E-ISAMY) (Megazyme, Kilruddery, Ireland) was employed to debranch purified rice starch. Subsequently, the relative molecular weight distribution was assessed using gel permeation chromatography (GPC) on a PL-GPC 220 system (Polymer Laboratories Varian Inc., Amherst, MA, USA), following the method described by Zhang et al. [15]. The GPC data were analyzed using integral equations to construct molecular weight distribution curves, employing standard dextran samples with molecular weights of 2800, 18,500, 111,900, 410,000, 1,050,000, 2,900,000, and 6,300,000. To facilitate comparison among amylopectin (AP), its short chains (AP1), long chains (AP2), and amylose (AM), two replicate measurements were performed.

### 2.8. Crystalline Structure Analysis

Crystalline structure analysis was performed to examine the supramolecular structure of rice starch. X-ray powder diffraction (XRD) measurements were conducted using a D8 ADVANCE X-ray diffractometer (Bruker AXS, Karlsruhe, Germany), and the relative crystallinity was quantified according to the methodology outlined by Zhang et al. [15]. Additionally, to explore the short-range molecular order near the surface of starch granules, Fourier-transform infrared spectroscopy (FTIR) was employed using a Varian 7000 FTIR spectrometer (PerkinElmer, Wellesley, MA, USA), following the approach described by Zhang et al. [15]. Absorbance values at 1047 cm^−1^ and 1022 cm^−1^ were extracted and corrected from the FTIR spectra.

### 2.9. RNA Extraction and Quantitative Reverse Transcriptase PCR Analysis

Total RNAs were extracted from the developing seeds 15 days after flowering (DAF) using the RNAprep Pure Plant Kit (Tiangen, Beijing, China), in accordance with the instructions provided by the manufacturer. The total RNA was reverse-transcribed using oligo (dT18) primers as per the protocol of the Vazyme reverse transcription kit (Vazyme, Nanjing, China). Quantitative reverse transcriptase PCR (RT-qPCR) analyses were performed using SYBR Green Real-Time PCR Master Mixes (Thermo Fisher Scientific, Waltham, MA, USA). For each sample, biological triplicates were prepared, and each biological replicate was further subjected to technical triplicates to ensure precision. The expression level of each gene was quantified by averaging the value obtained from these replicates.

### 2.10. Enzyme Activity Assays

The enzyme activity of granule-bound starch synthase I (GBSSI) was assessed according to the methodology delineated by Liu et al. [16]. Seeds harvested at 15 DAF were deprived of hull, embryo, and pericarp and subsequently ground into a fine powder under liquid nitrogen. The activity of GBSSI was quantitatively defined as the amount of NADPH generated per minute per gram of the sample, with one unit of enzyme activity being equivalent to 1 nmol of NADPH.

### 2.11. Western Blot Assay

For the Western blot analysis, immature seeds collected at 15 DAF were pulverized in liquid nitrogen to obtain a fine powder. Protein extraction was achieved by homogenizing the seed powder with an extraction buffer consisting of 125 mmol/L Tris-HCl, pH 6.8, 4 mol/L urea, 4% SDS, and 5% 2-mercaptoethanol at a ratio of 1:15 (15 μL of buffer per 1 mg of powder), followed by incubation at 37 °C for 3 h. The extracted proteins were then separated via SDS−PAGE and electrotransferred onto polyvinylidene difluoride (PVDF) membranes. Subsequent incubation steps were performed using antibodies specifically targeting GBSSI and HSP to detect the respective proteins.

### 2.12. Statistical Analysis

For sample characterization, RVA, GPC, XRD, and attenuated total reflectance–Fourier-transform infrared spectroscopy (ATR-FTIR) analyses were each conducted in duplicate. Other tests were performed independently in triplicate, with results reported as mean ± standard deviation. Statistical analysis was carried out using one-way analysis of variance (ANOVA) and Tukey’s multiple comparison test, as well as Pearson’s bivariate correlations, all performed using SPSS 21.0 software. Results were considered statistically significant at a probability value of *p* < 0.05.

## 3. Results

### 3.1. Effects of Two Functional Single Nucleotide Polymorphisms on Seed Transparency

Agronomic trait evaluation revealed uniformity in plant architecture and grain dimensions across the transgenic lines, yet unveiled disparities in grain transparency (Figure 1C and Figure S2). Intricately, Nip(*wx*)-*Wx^mq^* grains displayed a notably darker endosperm compared to Nip(*wx*)-*Wx^mp^*. The Wx^b−5c^ line, while showing a subtly darker endosperm relative to Nip (harboring the *Wx^b^* allele), demonstrated a clear enhancement in grain transparency against Nip(*wx*)-*Wx^mp^*. A quantitative analysis of grain transparency mirrored this observation, unveiling a progressive transparency gradient following the sequence: Nip(*wx*) < Nip(*wx*)-*Wx^mq^* < Nip(*wx*)-*Wx^mp^* < Nip(*wx*)-*Wx^b−5c^* < Nip(*Wx^b^*) (Figure 1D). This gradation underscores the intricate link between grain transparency and the presence of microscopic voids within the endosperm’s starch granules. To further elucidate the microstructural characteristics of rice grains, the cross-sections of their kernels were further examined using an SEM. This exploration revealed a distinct architecture of starch granules in the Nip(*Wx^b^*) endosperm, which is compactly and symmetrically organized, devoid of any discernible voids. In contrast, the starch granules of the other four rice variants were marred by the presence of conspicuous, diminutive cavities, the extent and frequency of which varied markedly across the samples. Particularly, the cavities within the starch granules from Nip(*wx*)-*Wx^mp^* rice were notably larger compared to those in Nip(*wx*)-*Wx^b−5c^*. Meanwhile, the quantity and dimensions of the cavities within the starch granules of Nip(*wx*)-*Wx^mq^* bore a resemblance to those observed in Nip(*wx*) (Figure 1E). These intragranular voids have been demonstrated to influence the transparency of the grain, which is consistent with our observations of varying grain transparency across the transgenic rice lines [15].

### 3.2. Effect of the Distinct Transgenic Rice Lines on Rice Eating Quality

**Figure 2:**
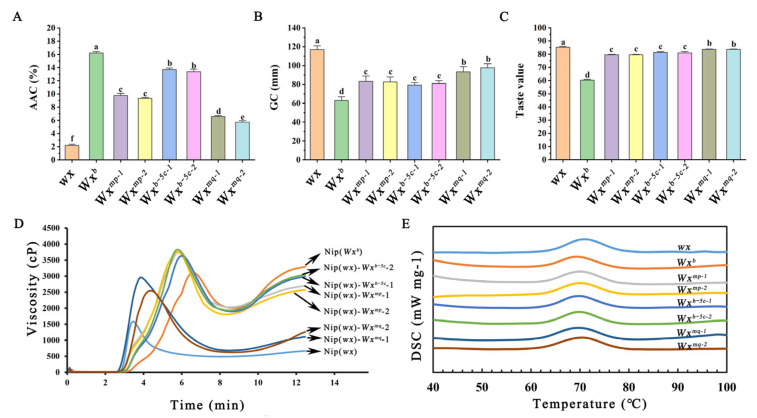
Analysis of grain physicochemical properties in transgenic rice lines: (**A**) Measures the amylose content (AC) across different transgenic lines. (**B**) Evaluates the gel consistency (GC) of rice, a parameter that reflects the viscous properties of rice gel, indicative of the texture and palatability of cooked rice. (**C**) Assesses the taste value of cooked rice. (**D**) Rapid Visco Analyzer (RVA) spectra of rice flours. (**E**) Gelatinization of rice flours analyzed using Differential Scanning Calorimetry (DSC). Statically significant differences are denoted by different letters (*p* < 0.01).

Our investigation extended to the analysis of the principal constituents and physicochemical characteristics of rice grain. Notably, the glutinous variety, Nip(*wx*), exhibited an exceptionally low AC of approximately 3%. In comparison, the Nip(*Wx^b^*) and Nip(*wx*)-*Wx^b−5c^* variants demonstrated a modest AC of about 13%, whereas the Nip(*wx*)-*Wx^mp^* line presented an AC of approximately 10%. The Nip(*wx*)-*Wx^mq^* line among the trio of transgenic lines emerged with the lowest AC of about 6% (Figure 2A). Subsequent assessments of GC, an indicator of texture, revealed that Nip(*wx*) was the softest, which was sequentially followed by Nip(*wx*)-*Wx^mq^*, Nip(*wx*)-*Wx^mp^*, Nip(*wx*)-*Wx^b−5c^*, and Nip(*Wx^b^*) (Figure 2B). These findings are consistent with the previously established inverse relationship between GC and AC [17]. Moreover, the softer GC of the transgenic rice lines underscored a marked enhancement in their culinary appeal. To quantify this enhancement, the taste value of the cooked rice was meticulously assessed. It emerged that all the transgenic variants transcended Nip(*Wx^b^*) in taste value, with Nip(*wx*)-*Wx^mq^* rice achieving a notably superior taste profile compared to both Nip(*wx*)-*Wx^mp^* and Nip(*wx*)-*Wx^b−5c^* rice (Figure 2C). This comprehensive analysis unveils the instrumental role of the functional mutations at Ex4-53A and Ex5-52C in modulating amylose synthesis, GC, and, ultimately, the taste value of cooked rice. The synergistic interplay between these mutations, particularly evident in the *Wx^mq^* allele, significantly accentuates the AC variation, enriching the culinary quality of these genotypes.

The viscosity of rice starch stands as another important indicator in the appraisal of rice’s culinary merit. To elucidate this aspect, we conducted an RVA analysis of flours derived from various transgenic rice lines. The RVA profiles, as shown in Figure 2D, revealed a considerable diversity across the samples. Specifically, the Nip(*wx*) rice exhibited a prototypical glutinous RVA trajectory, characterized by an early onset of gelatinization and the lowest final viscosity among the rice lines examined. It is noteworthy that the Nip(*wx*)-*Wx^mq^* rice mirrored the RVA pattern of Nip(wx) rice, albeit with a discernible shift towards the right, manifesting in elevated peak viscosity, hot paste viscosity, and final viscosity metrics. In addition, the RVA curves of Nip(*wx*)-*Wx^mp^* and Nip(*wx*)-Wx*^b−5c^* rice are situated between Nip(*Wx^b^*) and Nip(*wx*) rice. Despite the resemblance in their RVA curve contours, Nip(*wx*)-*Wx^mp^* rice distinguished itself with a marginally higher peak viscosity and breakdown values, albeit with a reduced final viscosity when compared with Nip(*wx*)-*Wx^b−5c^* rice (Table 1). These findings provide support for the hypothesis that rice varieties with reduced AC display a greater susceptibility to gelatinization, as evidenced by leftward shifts in their pasting curves. This observation concurs with our preceding investigations [15], further substantiating the correlation between AC and the role of starch viscosity in determining the culinary quality of rice.

Additionally, the gelatinization properties of rice flours were characterized using the DSC. The gelatinization curves obtained, as shown in Figure 2E, demonstrated a general uniformity across all samples, albeit with the glutinous Nip(*wx*) rice displaying a slight shift to the right in its curve. In terms of the gelatinization parameters, there was a consensus among the transgenic rice lines in terms of both onset temperature (T_o_) and peak temperature (T_p_); however, variations were observed in the enthalpy of gelatinization (ΔH_gel_) (Table 2). Previous research has shown that the enthalpy of starch gelatinisation reflects the thermal energy required to disrupt the semi-crystalline structure of starch molecules, predominantly composed of amylopectin [18]. It is generally accepted that increased amylopectin content correlates with higher crystallinity in rice starch. It is therefore plausible that the glutinous Nip(*wx*) rice, presumably with a higher amylopectin content, had the highest ΔHgel, followed in descending order by Nip(*wx*)-*Wx^mq^*, Nip(*wx*)-*Wx^mp^*, Nip(*wx*)-*Wx^b−5c^* and Nip(*Wx^b^*). These results are consistent with previously reported observations that demonstrate an inverse relationship between gelatinization enthalpy and AC in rice grains [15].

### 3.3. Comparison of Starch Crystalline Structure in Transgenic Rice

**Figure 3:**
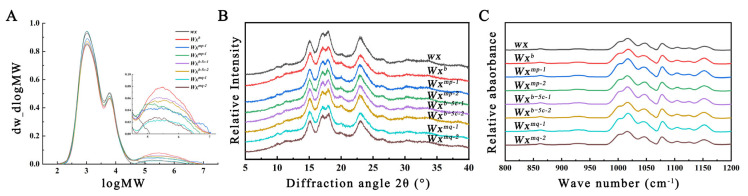
Crystalline structures of starch granules from different transgenic rice lines: (**A**) Gel permeation chromatograms of isoamylase-debranched starches. AP1, AP2, and AM indicate amylopectin short-branched chains (AP1), long-branched chains (AP2), and amylose (AM), respectively. (**B**) XRD patterns and (**C**) ATR-FTIR spectra of rice starches from different transgenic rice lines.

Rice starch is a semi-crystalline complex comprising amylopectin and amylose, with its physicochemical properties intricately linked to its fine structure. Analysis of the fine structure is pivotal to understanding the variances in taste and quality among different rice varieties. The relative molecular weight distribution of starch in various transgenic lines was assessed using high-temperature GPC. As shown in Figure 3A, the starch from Nip(*wx*) exhibited only two fractions: the branched short-chain component (AP1) and the branched long-chain component (AP2). In contrast, starch from other samples showed, in addition to two amylopectin peaks, a distinct amylose peak (AM). It has been shown that the AP1 component of amylopectin consists of low-molecular-weight amylopectin A and short B chains (A+B1 chains), while the AP2 component contains high-molecular-weight long B chains [19]. Significant variations in the amylopectin to amylose ratios were observed across different transgenic lines. Notably, the AM values in Nip(*wx*)-*Wx^mq^* differed compared to those in Nip(*wx*)-*Wx^mp^* and Nip(*wx*)-*Wx^b−5c^* (Table 3). GPC analysis identified the AM components as indicative of the actual straight-chain starch content within the starch matrix. This finding implies that both SNPs Ex4-53G/A and Ex5-53T/C can differentially reduce the presence of straight-chain starch in rice, albeit with varying effects. Moreover, the combined effect of these two genetic loci significantly amplifies the observed AC disparity.

The degree of long-range order in starch granules from various transgenic lines was quantitatively assessed and evaluated using XRD. All examined samples exhibited pronounced diffraction peaks around diffraction angles of 15°, 17°, 18°, and 23°, characteristic of the A-type crystalline structure typically observed in cereal starches. Although the diffraction curves of samples from different transgenic lines were similar, crystallinity calculations indicated highly significant disparities (Figure 3B, Table 3). Specifically, the Nip(*wx*) sample demonstrated the highest crystallinity, followed by Nip(*wx*)-*Wx^mq^*, with Nip(*Wx^b^*) exhibiting the lowest. Previous studies have indicated a negative correlation between starch crystallinity and AC levels [20], and higher crystallinity levels necessitate more heat for the endothermic reaction [21], explaining the highest enthalpy value observed in Nip(*wx*) and the lowest in Nip(*Wx^b^*).

Further investigation of the short-range order of starches from the grains of different transgenic lines was conducted using ATR-FTIR. The results indicated that all samples yielded similar deconvoluted FTIR spectra. The analysis of parameters (1045/1022 cm^−1^) revealed that the degree of short-range order was the highest in the Nip(*wx*) sample, followed by Nip(*w*x)-*Wx^mq^*, with the lowest observed in Nip(*Wx^b^*) (Figure 3C, Table 3). These findings are consistent with the XRD analysis and corroborate our prior studies on the crystal structure of rice starch [15].

### 3.4. Effects of Two Functional SNPs on Wx Gene Expression and GBSSI Enzyme Activity

**Figure 4:**
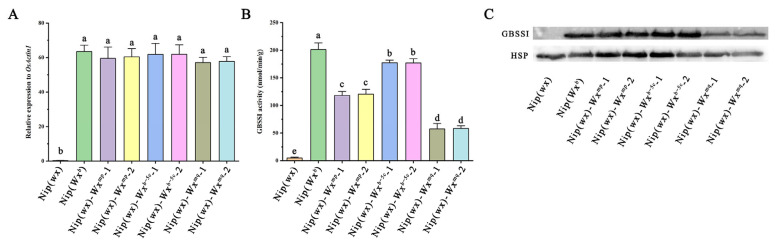
*Wx* allele functionality in transgenic rice lines:(**A**) Displays the relative expression levels of Wx gene in immature seeds. (**B**) Reports on the activity of granule-bound starch synthase I (GBSSI), the enzyme responsible for amylose synthesis in rice. (**C**) Illustrates the results of Western blot analysis for the GBSSI protein, visualizing the presence and abundance of this key enzyme in the transgenic rice lines, further elucidating the molecular underpinnings of starch synthesis regulation. Statically significant differences are denoted by different letters (*p* < 0.01).

To elucidate the impact of two functional mutations within the *Wx* gene on its expression and the activity of the GBSSI enzyme, we conducted a comparative analysis of *Wx* gene expression across three distinct transgenic lines. Gene expression was assessed in developing seeds at 15 DAF. The results revealed no significant variance in mRNA levels among the transgenic lines when compared to Nip(*Wx^b^*) rice. Notably, the glutinous Nip(*wx*) rice exhibited negligible expression (Figure 4A). These findings align with previous studies [10,15], suggesting that such SNP mutations do not substantially alter *Wx* gene expression.

Subsequent assessments of GBSSI enzyme activity revealed that Nip(*wx*)-*Wx^mq^* exhibited the lowest activity, followed by Nip(*wx*)-*Wx^mp^* and Nip(*wx*)-*Wx^b−5c^*, in comparison to Nip(*Wx^b^*) rice (Figure 4B). Western blotting analyses of GBSSI corroborated these findings, showing a progressive decrease in GBSSI abundance in the order of Nip(*Wx^b^*) > Nip(*wx*)-*Wx^b−5c^* > Nip(*wx*)-*Wx^mp^* > Nip(*wx*)-*Wx^mq^* > Nip(*wx*), (Figure 4C) suggesting that the mutations not only reduce GBSSI enzyme activity but also impair its starch-binding capacity. Consequently, both the Ex4-53A and Ex5-52C mutations were observed to decrease GBSSI enzyme activity and abundance, leading to a reduction in AC in rice endosperm. Additionally, the results indicated that the Ex5-52C mutation exerts a minor effect on AC compared to the Ex4-53A mutation, suggesting its potential utility in rice breeding for optimizing grain transparency and culinary quality.

## 4. Discussion

In recent years, rice varieties with low AC, typically ranging from 8% to 12% and referred to as soft rice, have gained popularity across China due to their superior culinary qualities. Notably, the soft rice varieties from the Nangeng series, which carry the *Wx^mp^* allele, have achieved extensive cultivation in Jiangsu province, yielding significant economic gains [7]. Besides the *Wx^mp^* and *Wx^mq^* alleles, the *Wx^op/hp^* alleles, predominantly found in *indica* rice varieties, are also recognized for their contributions to lowering AC [11,22]. However, in the context of *japonica* soft rice breeding programs within China, the *Wx^mp^* allele has been the preferred choice. The *Wx^mq^* allele, despite its potential, has often been overlooked or confused with *Wx^mp^*, possibly due to its significant impact on reducing AC. Prior research posited that the mutation at Ex4-53A could play a pivotal role in influencing AC in both *Wx^mp^* and *Wx^mq^* alleles, although experimental validation has been limited [10]. Our research has demonstrated that the Ex4-53A mutation exerts a more pronounced effect on AC and various grain quality attributes than the Ex5-52C mutation within the *Wx^b^* genetic background. Furthermore, the simultaneous mutations at both the Ex4-53A and Ex5-52C sites (*Wx^mq^* allele) resulted in the lowest AC among the evaluated transgenic rice lines. Our study delineates the distinct impacts of the Ex4-53A and Ex5-52C mutations on the quality profile of rice grains and demonstrates their potential application in future high-quality rice breeding.

The appearance quality of rice mainly involves grain transparency and chalkiness, with AC playing a crucial role in determining grain transparency. Varieties such as glutinous rice, which have an extremely low AC, exhibit an opaque waxy phenotype. Conversely, soft rice varieties with low AC often display a semi-transparent or cloudy opaque endosperm phenotype. Previous research has demonstrated a significant negative correlation between the number and size of pores within the starch granules of rice endosperm and the transparency of the rice [15,23]. This study corroborates these findings, observing a similar pattern across three distinct transgenic rice lines and the Nip(*wx*) endosperm, where large pores within the starch granules were particularly prominent in glutinous rice. The opaque endosperm phenotype, externally noticeable in soft rice varieties with low AC, constitutes a major defect as lower AC correlates with decreased grain transparency. Thus, the transparency of Nip(*wx*)-*Wx^mq^* is relatively lower compared to Nip(*wx*)-*Wx^mp^* and Nip(*wx*)-*Wx^b−5c^* rice, aligning with its lower AC feature.

From the perspective of improving the appearance quality of rice, efforts to lower AC to improve flavor have led to the undesirable effect of reduced grain transparency. Recent studies have demonstrated the feasibility of creating novel *Wx* alleles to regulate AC, suggesting that genetic editing of cis-regulatory elements in the *Wx* promoter could finely tune AC, thereby impacting transparency [24,25]. In addition, nucleotide editing at sites responsible for low AC alleles at the N-terminus of the GBSSI has resulted in a series of mutants with AC ranging from 1.4% to 11.9% [26], while editing nucleotides in the middle region of GBSSI has generated a series of new *Wx* alleles with AC levels ranging from 0.3% to 29.43% [27]. From the perspective of improving the visual quality of rice, lowering AC to improve the flavor of rice has resulted in a defect of poor grain transparency. Therefore, to cultivate rice that excels in both taste and visual quality, moderately adjusting AC is a viable strategy to balance these attributes. Moreover, studies indicate that modifying the fine structure of amylopectin can enhance the transparency of the endosperm [28]. Therefore, expanding rice genetic resources or utilizing gene editing technologies holds great promise for addressing the problem of opaque endosperm in low-AC rice varieties. It is worth noting that the discovery of the Ex5-52C mutation in this research marks a significant advancement, providing a moderate level of AC that effectively balances optimal grain transparency with ideal nutritional quality.

Our findings have significant implications for rice breeding programs focused on enhancing the cooking and appearance qualities of rice varieties. By elucidating the impact of specific SNP mutations in the *Wx* gene on AC and grain quality traits, we provide breeders with valuable insights to selectively target desirable alleles in their breeding efforts. The identification of key mutation sites such as Ex4-53A and Ex5-52C facilitates precise genetic modifications, enabling breeders to finely tune AC levels while maintaining other favorable traits. In addition, the development of novel *Wx* alleles through gene editing opens avenues for crafting rice varieties with tailored AC levels, aligning with consumer preferences for both taste and visual appeal.

## Figures and Tables

**Table 1:** Pasting properties of rice flours from transgenic rice and its wild type.

Line	Peak Viscosity (cP)	Trough Viscosity (cP)	Breakdown Viscosity (cP)	Final Viscosity (cP)	Setback Viscosity (cP)
Nip(*wx*)	1575.50 ± 17.68 g	495.00 ± 9.90 f	1116.00 ± 22.63 f	660.00 ± 9.90 g	−927.50 ± 9.19 b
Nip(*Wx^b^*)	3109.00 ± 11.31 d	1792.50 ± 6.36 c	1307.00 ± 18.38 e	3235.00 ± 16.97 a	135.50 ± 7.78 a
Nip(*wx*)-*Wx^mp^*-1	3774.00 ± 18.38 a	1815.50 ± 10.60 c	1963.00 ± 14.14 b	2558.50 ± 14.85 d	−1185.00 ± 9.90 e
Nip(*wx*)-*Wx^mp^*-2	3762.50 ± 10.61 a	2014.50 ± 19.09 a	1954.00 ± 16.97 bc	2680.50 ± 19.09 c	−1089.50 ± 19.09 d
Nip(*wx*)-*Wx^b−5c^*-1	3568.00 ± 18.19 c	1672.00 ± 18.38 d	1904.00 ± 15.56 cd	2577.00 ± 14.14 d	−1004.00 ± 24.04 c
Nip(*wx*)-*Wx^b−5c^*-2	3654.00 ± 24.49 b	1974.50 ± 19.09 b	1886.50 ± 20.51 d	2796.00 ± 24.04 b	−1032.50 ± 20.51 c
Nip(*wx*)-*Wx^mq^*-1	2970.50 ± 10.60 e	667.00 ± 19.80 e	2262.50 ± 27.58 a	1124.00 ± 26.87 f	−1845.00 ± 18.38 g
Nip(*wx*)-*Wx^mq^*-2	2532.50 ± 16.26 f	636.50 ± 16.26 e	1935.50 ± 23.33 bcd	1251.50 ± 16.26 e	−1295.00 ± 19.80 f

Data represent means ± standard deviations, *n* = 2. For each column in the same flour or starch samples, values displaying different lowercase letters are significantly different by one-way analysis of variance (ANOVA) with Tukey’s multiple comparisons (*p* < 0.05).

**Table 2:** Thermal properties of rice flours from transgenic rice and its wild type.

Line	T_o_ (°C)	T_p_ (°C)	T_c_ (°C)	ΔH (J·g^−1^)
Nip(*wx*)	61.55 ± 0.21 c	69.30 ± 0.14 c	77.15 ± 0.14 c	7.45 ± 0.05 a
Nip(*Wx^b^*)	63.85 ± 0.35 a	71.05 ± 0.21 a	78.65 ± 0.21 a	5.90 ± 0.10 d
Nip(*wx*)-*Wx^mp^*-1	62.95 ± 0.35 b	70.05 ± 0.07 b	77.55 ± 0.21 bc	6.48 ± 0.08 c
Nip(*wx*)-*Wx^mp^*-2	63.00 ± 0.28 b	70.15 ± 0.07 b	77.30 ± 0.14 c	6.50 ± 0.06 c
Nip(*wx*)-*Wx^b−5c^*-1	62.80 ± 0.14 b	70.00 ± 0.28 b	77.45 ± 0.07 bc	6.48 ± 0.06 c
Nip(*wx*)-*Wx^b−5c^*-2	62.95 ± 0.35 b	70.00 ± 0.14 b	77.40 ± 0.28 bc	6.49 ± 0.03 c
Nip(*wx*)-*Wx^mq^*-1	62.65 ± 0.35 b	70.05 ± 0.35 b	77.65 ± 0.21 bc	7.25 ± 0.03 b
Nip(*wx*)-*Wx^mq^*-2	62.92 ± 0.07 b	70.30 ± 0.14 b	77.85 ± 0.21 b	7.24 ± 0.04 b

The data represent means ± standard deviation, *n* = 3. To, Tp, Tc, and ΔH indicate onset temperature, peak temperature, conclusion temperature, and enthalpy of gelatinization, respectively. Means with different lowercase letters in each column for the same cultivar are significantly different by one-way analysis of variance (ANOVA) with multiple comparisons (*p* < 0.05).

**Table 3:** GPC, XRD, and ATR-FTIR parameters from transgenic rice and its wild type.

Line	AP1	AP2	AM	Relative Crystallinity (%)	1045/1022 cm^−1^
Nip(*wx*)	74.22 ± 0.29 a	25.78 ± 0.29 a	-	29.6 ± 0.13 a	0.64 ± 0.006 a
Nip(*Wx^b^*)	63.52 ± 0.3 e	22.24 ± 0.09 d	14.24 ± 0.21 a	26.6 ± 0.28 e	0.55 ± 0.004 e
Nip(*wx*)-*Wx^mp^*-1	69.42 ± 0.23 c	23.5 ± 0.37 c	7.08 ± 0.13 c	28.24 ± 0.14 c	0.6 ± 0.002 c
Nip(*wx*)-*Wx^mp^*-2	69.22 ± 0.28 c	23.59 ± 0.11 c	7.19 ± 0.17 c	28.22 ± 0.11 c	0.6 ± 0.004 c
Nip(*wx*)-*Wx^b−5c^*-1	66.39 ± 0.01 d	23.16 ± 0.12 c	10.46 ± 0.11 b	27.53 ± 0.33 d	0.57 ± 0.003 d
Nip(*wx*)-*Wx^b−5c^*-2	65.82 ± 0.4 d	23.42 ± 0.22 c	10.76 ± 0.18 b	27.46 ± 0.24 d	0.58 ± 0.004 d
Nip(*wx*)-*Wx^mq^*-1	71.82 ± 0.35 b	24.48 ± 0.3 b	3.7 ± 0.05 d	28.82 ± 0.11 b	0.63 ± 0.004 b
Nip(*wx*)-*Wx^mq^*-1	72.17 ± 0.42b	24.52 ± 0.33 b	3.31 ± 0.1 e	28.82 ± 0.2 b	0.63 ± 0.003 b

Data represent means ± standard deviations, *n* = 2. For each column in the same flour or starch samples, values displaying different lowercase letters are significantly different by one-way analysis of variance (ANOVA) with Tukey’s multiple comparisons (*p* < 0.05). AP1, amylopectin short-branched chains; AP2, long-branched chains; AM, amylose.

## Data Availability

The data presented in this study are available on request from the corresponding author. The data are not publicly available due to privacy restrictions.

## Supplementary Materials

The following supporting information can be downloaded at: https://www.mdpi.com/article/10.3390/foods13111624/s1, Figure S1: A schematic diagram of *Wx* gene structure and the primers used for plasmid construction, Figure S2: The phenotype of the transgenic rice plants, Table S1: Primers used in this study.
