# Supplementary material for: Deciphering the Role of Waxy Gene Mutations in Enhancing Rice Grain Quality
Source: Foods. 2024 May 23;13(11):1624. doi: 10.3390/foods13111624 (PMC11171567; doi:10.3390/foods13111624)
Supplement: Supplementary file 1 [file foods-13-01624-s001.zip › foods-2991862-supplementary.pdf]

foods

Table S1. Primers used in this study.

Name	Primers (5'-3')	Purpose
QWx-1	AAGCTTACTTAGTCTCGATCTGACGTGGTG	Gene amplification, sequencing, and construction of transgenic vectors
QWx-2	CGCCTGCAAAGAACACAAGAACACAACATT	
QWx-3	AACAATTCAATTCAGTGCAGAGATCTTCCA	
QWx-4	CTCCACAGCCATAAGCCACACCAACT	
QWx-5C-F	CACTGGAGTTGATCACAAAGACAACC	
QWx-5C-R	GGTTGTCTTTGTGATCAACTCCAGTG	
maWx-F	ATTCCTTCAGTTCTTTGTCTATCTCA	RT-qPCR detection
maWx-R	ATGGTGGTTGTCTAGCTGTTGC	
Actin01-F	CCAAGGCCAATCGTGAGAAGA	
Actin01-R	AATCAGTGAGATCACGCCCAG	
SPS-F	TTGCGCCTGAACGGATAT	RT-qPCR detection of *Wx* copy number
SPS-R	CGGTTGATCTTTTCGGGATG	
WxN-F	AACTGGATGAAGGCCGGAAT	
WxN-R	TGTACTTGTCCTTGCTGGGAT	

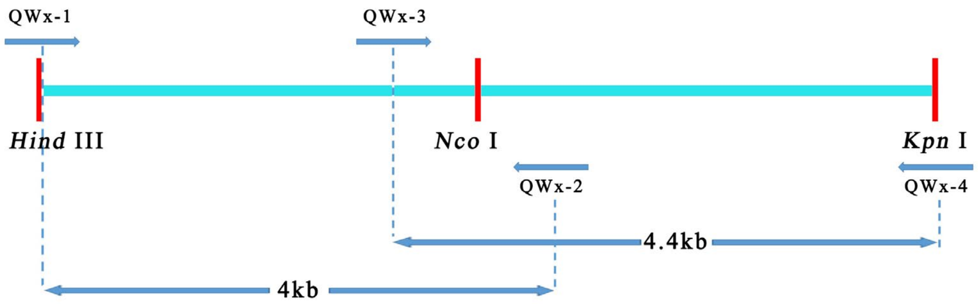

Figure S1. The schematic diagram of *Wx* gene structure and the primers used for plasmids construction.

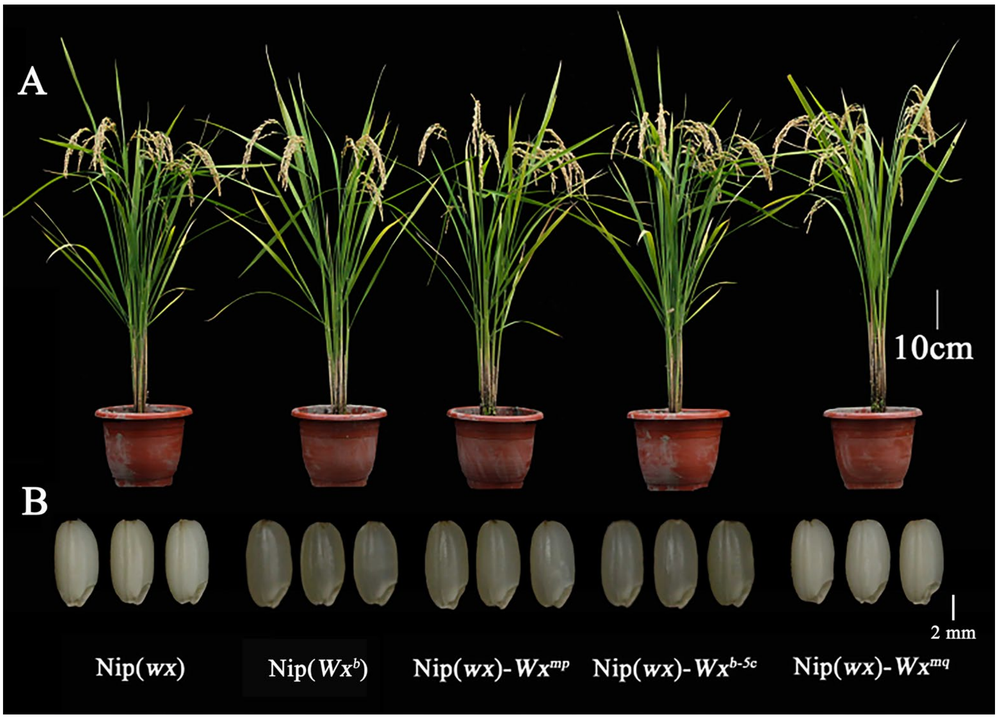

Figure S2. Phenotype of the transgenic rice plants. (A) Phenotypes of mature plants from different transgenic lines. (B) Phenotypes of brown rice from different transgenic lines.
